# Milk-Borne Epidemics in Greater Boston

**Published:** 1914-12

**Authors:** 


**Milk-Borne Epidemics in Greater Boston.** M. J. Rosenau, in his book on the Milk Question, gives the following statistics: 1907, Diphtheria, 72 cases; 1908, Scarlet fever, 717; 1909, Typhoid, 400; 1910, Scarlet fever, 842; 1911, Amygdalitis, 2,064; Total, 4,095.

